# Effects of coronal implant neck flutes on early peri-implant osteogenesis and stability

**DOI:** 10.1186/s40729-026-00674-6

**Published:** 2026-04-17

**Authors:** Ziqi Xie, Toru Ogawa, Kenta Shobara, Nobuhiro Yoda

**Affiliations:** 1 Division of Advanced Prosthetic Dentistry, Tohoku University Graduate School of Dentistry, Sendai, Japan; 2 Division of Comprehensive Dentistry, Tohoku University Graduate School of Dentistry, Sendai, Japan

**Keywords:** Dental implants, Osseointegration, Implant stability, Implant neck design

## Abstract

**Purpose:**

Limited research has investigated how implant neck flute design influences early peri-implant osteogenesis and implant stability in vivo. This study evaluated the effects of different neck flute designs on peri-implant bone healing and osseointegration in rat tibiae.

**Methods:**

Three types of custom-made implants (13 mm total length, 8 mm screw part, 2 mm diameter) were used: Type A (4 coronal flutes, 1.50 mm length, 0.2 mm notch radius, 0.20 mm depth), Type B (4 coronal flutes, 1.50 mm length, 0.5 mm notch radius, 0.20 mm depth), and Type C (no coronal flutes). The implants were bilaterally inserted into the tibiae of 21 male Wistar rats. After 4 weeks of healing, removal torque testing, micro-computed tomography, histological, and histomorphometric assessments were performed to evaluate osseointegration and the quality and quantity of the peri-implant bone. The assessments included the relative gray value (RGV; water = 0, implant = 100), bone-to-implant contact (BIC), and peri-implant bone volume/tissue volume (BV/TV).

**Results:**

Removal torque tests demonstrated significant differences among implant types, with Type A and Type B implants exhibiting higher values than Type C. The RGV revealed no significant differences among groups. Histomorphometric analysis revealed no significant differences among groups.

**Conclusion:**

The coronal flute design was associated with improved implant stability and osseointegration compared to non-fluted implants. This suggests that neck flute designs may enhance early peri-implant osseointegration and stability.

## Background

Dental implants are widely recognized as a reliable treatment option for the oral rehabilitation of patients with partial or complete edentulism, offering stable support for various types of prostheses [1, 2].

Osseointegration, originally defined by Brånemark in 1977 as the direct structural and functional connection between the bone and implant surface, is a key determinant of long-term clinical success [3–5]. Implant stability is a crucial factor for osseointegration [6, 7]. Stability depends on the direct mechanical connection between the implant surface and surrounding bone, and can be divided into primary and secondary stability [8, 9]. Primary stability is achieved by mechanical fixation of the implant with the bone [9], whereas secondary stability is achieved through bone remodeling and maturation at the implant–bone interface [3]. Considering the critical role of stability in implant success, various design parameters, including the implant shape, surface properties, and thread design, have been explored.

For example, increasing the implant length enlarges the bone–implant contact area, thereby improving initial stability and supporting osseointegration [10]. Likewise, macro-irregularities such as grooves and pores of different dimensions have been incorporated into implant threads to enhance initial bone contact, increase surface area, and dissipate interfacial stress [11, 12]. However, a universally accepted “ideal” implant design has yet to be established [13].

Therefore, further innovations in implant macrogeometry are essential, particularly to enhance peri-implant osteogenesis. The implant neck, a transitional zone between the prosthetic structure and the alveolar bone, has increasingly been recognized as a critical site for initiating bone–implant interactions [14]. Previous studies on rat tibiae have demonstrated a strong osteogenic response around the upper implant neck, with the peri-implant bone exhibiting more prominent osteogenesis on the apical side [15]. These findings highlight the necessity of improving osteogenesis specifically around dental implant necks.

Studies have shown that blood clots formed on dental implant surfaces act as biological scaffolds.

These scaffolds facilitate the recruitment and differentiation of bone marrow mesenchymal stem cells (BMSCs) into osteoblasts, which are essential for bone formation. Furthermore, this process initiates a cascade of biological events, including activation of the coagulation system and cell adhesion, which are vital for osseointegration and bone healing [16, 17].

The neck flute design represents a novel macro-level modification of dental implants, featuring a unique fluted structure around the neck. This innovative design is hypothesized to enhance surface area for bone contact and optimize blood flow, thereby promoting blood clot formation at the implant neck. Consequently, it can support the biological processes essential for bone healing, improve implant stability, and enhance bone integration at the implant neck.

The aim of this study was to evaluate the hypothesis that the neck flute design improves bone healing, osseointegration at the implant neck, and implant stability. Ultimately, the goal was to determine its potential clinical application in patients.

## Materials and methods

### Animals

Experiments were conducted using 21 male Wistar rats (age: 12 weeks; average weight: 267.8 g). The rats were kept under climate-controlled conditions (23.5 °C, 50% humidity, 12-hour light/dark cycle) with free access to a standard laboratory diet and tap water.

The study was approved by the Institutional Animal Care and Use Committee of the Tohoku University Environmental & Safety Committee and was conducted at the Institute for Animal Experimentation at Tohoku University Graduate School of Medicine.

To minimize the number of experimental animals, the sample size per group (*n* = 7) was determined using a significance level of 5% and a statistical power of 80%. The calculation assumed an expected 25% difference between the groups and an anticipated standard deviation of 15%. These parameters were informed by the primary outcome measures (e.g., bone-to-implant contact [BIC] and removal torque) reported in previous studies employing a similar rat tibial implant model [15].

### Experimental design

**Fig. 1:**
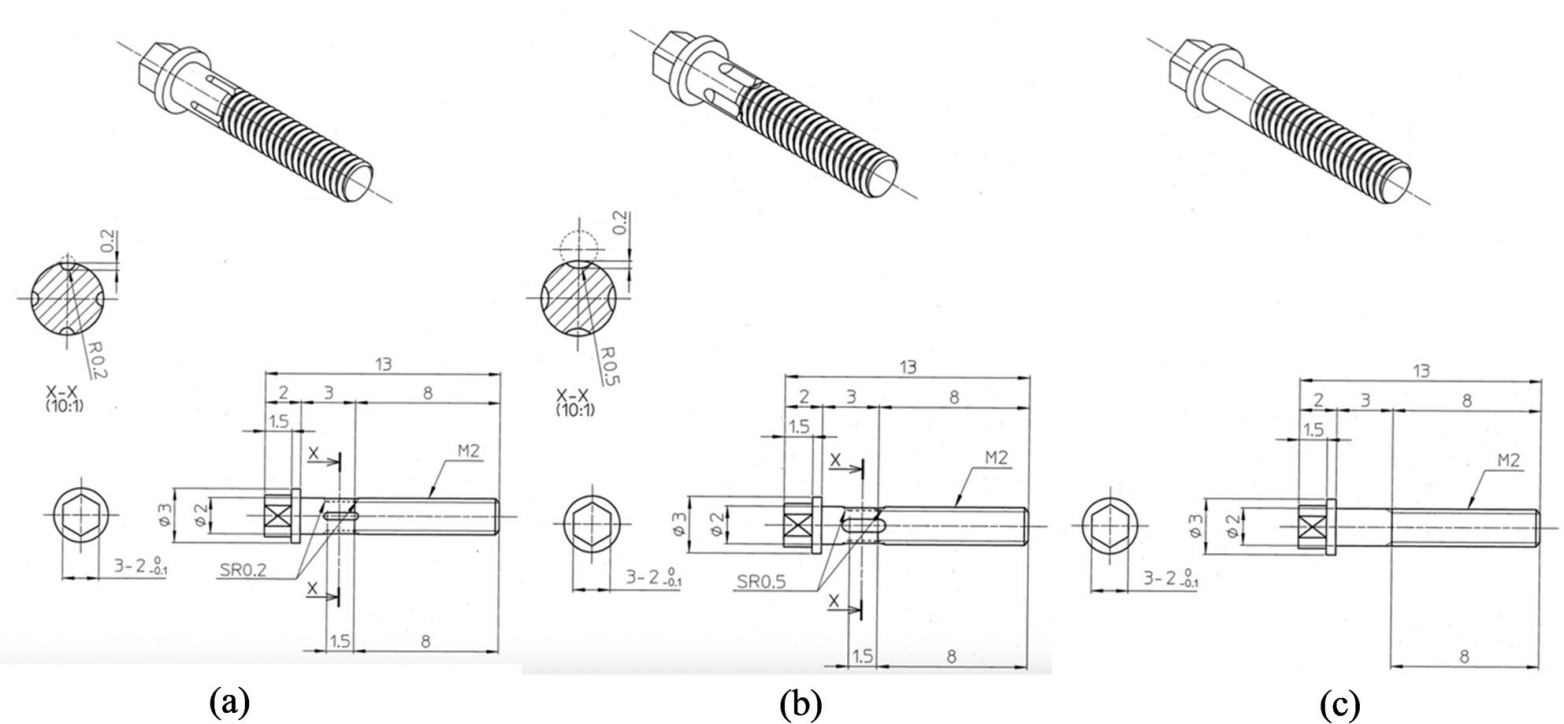
Three types of custom-made implant designs. **a** Type A implant; **b** Type B implant; **c** Type C implant

Three types of custom-made implants (13 mm total length, 8 mm screw portion, 2 mm diameter) were used: Type A (4 coronal flutes, 1.50 mm length, 0.2 mm notch radius, 0.20 mm depth), Type B (4 coronal flutes, 1.50 mm length, 0.5 mm notch radius, 0.20 mm depth), and Type C (no coronal flutes) (Fig. 1a, b, c). All implants were fabricated from commercially pure titanium with machined surfaces and treated with ultrasonic cleaning and alcohol rinsing.

**Fig. 2:**
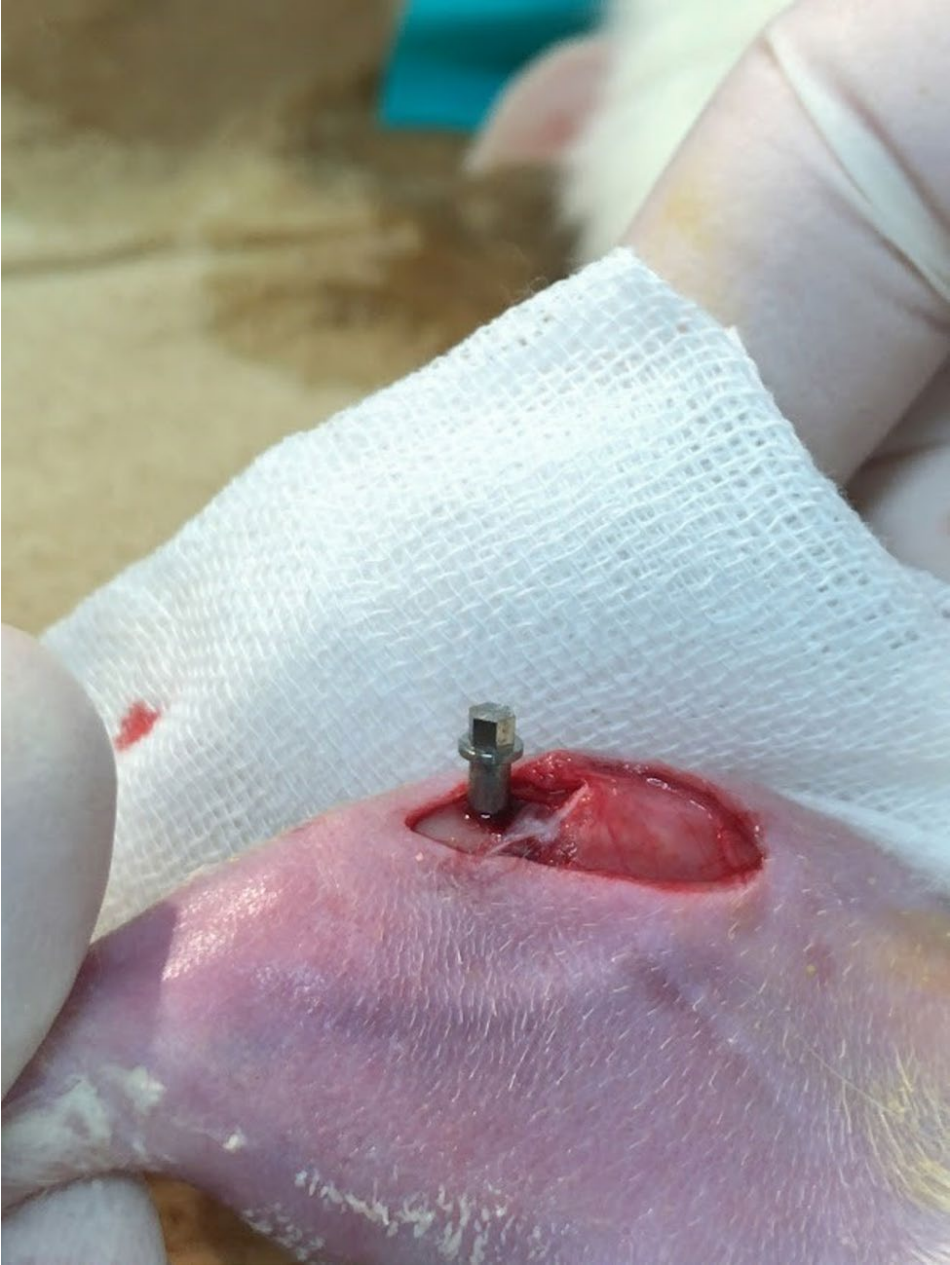
Location of implant placement, approximately 10 mm distal to the knee joint. This represents the surgical placement of the Type C implant

Twenty-one rats were randomly divided into three groups (*n* = 7): Type A, B, and C implant groups. Implants were inserted into the proximal metaphysis of both tibiae in all groups. Surgery was performed under anesthesia (2.5% isoflurane; Escain, Mylan, Pittsburgh, PA, USA) under aseptic conditions. A skin incision was made on the medial side of the tibia, and both cortices were perforated with a surgical drill at a low rotational speed under constant saline cooling (Implant Motor IM-III, GC, Japan). Each implant was placed approximately 10 mm distal to the knee joint (Fig. 2). Implants were placed in a partially submerged manner: the apical portion was inserted into the cortical bone, while the fluted neck region remained partially exposed above the bone surface. This placement allowed evaluation of bone healing and osseointegration at the implant neck. Implants were inserted using a custom-fit manual torque wrench. Following implant placement, surgical wounds were closed using 5–0 polyglycolic acid sutures (Matsuda Ika Kogyo Co., Ltd., Japan).

All rats were euthanized weeks after implantation under isoflurane-induced anesthesia. The tibiae with the implants were subsequently dissected. One tibia from each rat underwent a removal torque test to assess the biomechanical strength of osseointegration, while the contralateral tibia was subjected to micro-CT), histological, and histomorphometric assessments to evaluate the quality and quantity of the peri-implant bone and osseointegration.

### Evaluation of implant osseointegration

#### Removal torque test

The tibia was secured using a jig to ensure that the long axis of the implant was perpendicular to the fixation base. A torque gauge (ATG1.5CN/ATG12CN; Tohnichi Mfg, Tokyo, Japan) was attached to the implant head, and a horizontal rotational load was applied in the opposite direction of the rotational force used during implant placement. The removal torque (RT) value was defined as the maximum rotational load required to rotate the implant horizontally.

#### Micro-CT analysis

A micro-CT test was performed using a micro-CT system (ScanXmate-D225RSS270; Comscantecno, Kanagawa, Japan), with parameters set at 200 kV and 100 µA. Following three-dimensional reconstruction, a sagittal slice along the axis of the tibia and dental implant, passing through the implant center was selected for analysis.

**Fig. 3:**
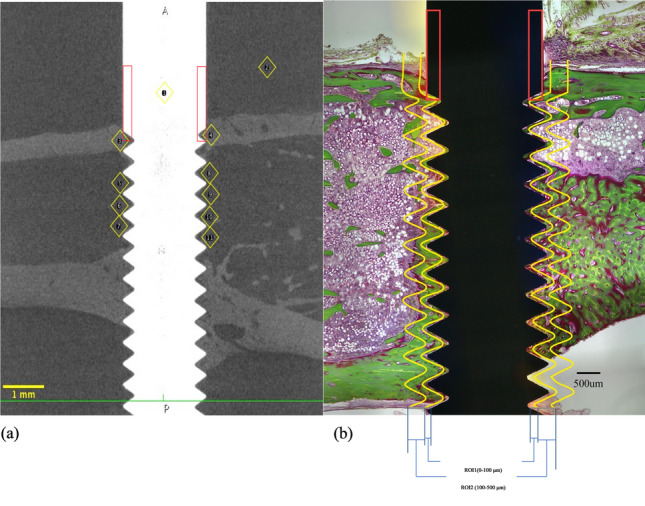
Definition of regions of interest (ROI) for micro-computed tomography (CT) and histomorphometric analysis. **a** Region of interest (ROI) for the micro-CT analysis. ROI comprises 0.3 × 0.3 mm regions adjacent to the implant neck surface, respectively. **b** Reference sites for bone volume relative to tissue volume (BV/TV). ROI1: 0–100 μm from the implant surface; ROI2: 100–500 μm from the implant surface. The red rectangle marks the presumed location of the flute at the implant–bone interface, which was not captured in this section

The relative gray value (RGV) was evaluated using the ImageJ2 software (version 2.14.0/1.54f; National Institutes of Health, Bethesda, MD, USA). Measurements were obtained from a 0.3 × 0.3 mm region of interest (ROI) set in the peri-implant cortical and cancellous bone. The RGV of the cortical bone and cancellous bone was calculated using water and the implant as internal reference standards with their gray values set at 0 and 100, respectively (Fig. 3a).

#### Histological and histomorphometric analysis

Following micro-CT analysis, the bone–implant blocks were fixed in phosphate-buffered formalin solution and dehydrated through a graded series of increasing concentrations of alcohol. After dehydration, the samples were embedded in poly (methyl methacrylate). Embedded samples were sectioned along the tibial and implant axes using a diamond saw (Exakt BS-300CP; Exakt Technologies, Norderstedt, Germany). Sections were then polished to a final sample thickness of 40 μm (Exakt MG-400CS; Exakt Technologies)and stained with Villanueva–Goldner stain.

Histological and histomorphometric analyses were performed using a light microscope at ×40 magnification (Leica LMD7000; Leica Microsystems, Wetzlar, Germany). The samples were scanned using a high-sensitivity camera (Leica DFC295; Leica Laborlux). Histomorphometric analysis was performed via digital imaging using Adobe Photoshop (version 23.5.2; Adobe Systems, San Jose, CA, USA) and ImageJ2 software (version 2.14.0/1.54f; National Institutes of Health, Bethesda, MD, USA). The following analyses were performed:

(i) BIC (%): (Calculated as the total length of direct bone–implant contact (µm) / implant length extending from the most medial to most lateral BIC point (µm)) × 100 (Fig. 3b).
(ii) Peri-implant bone volume relative to tissue volume (BV/TV; %): (Calculated as the bone area (µm^2^) / reference area (µm^2^)) × 100. Two ROI were defined: 0–100 μm (BV/TV ROI1) and 100–500 μm (BV/TV ROI2) zones extending from the implant surface (Fig. 3b).

### Statistical analysis

Data were expressed as mean ± standard deviation (SD). Statistical analyses were performed using the JMP software (version Pro 17; SAS Institute Inc., Cary, NC, USA). Group differences were evaluated with the Wilcoxon rank-sum test and Kruskal–Wallis test. Dunn’s method was used for post-hoc comparisons. Statistical significance was set at *p* < 0.05.

## Results

### Removal torque test

**Fig. 4:**
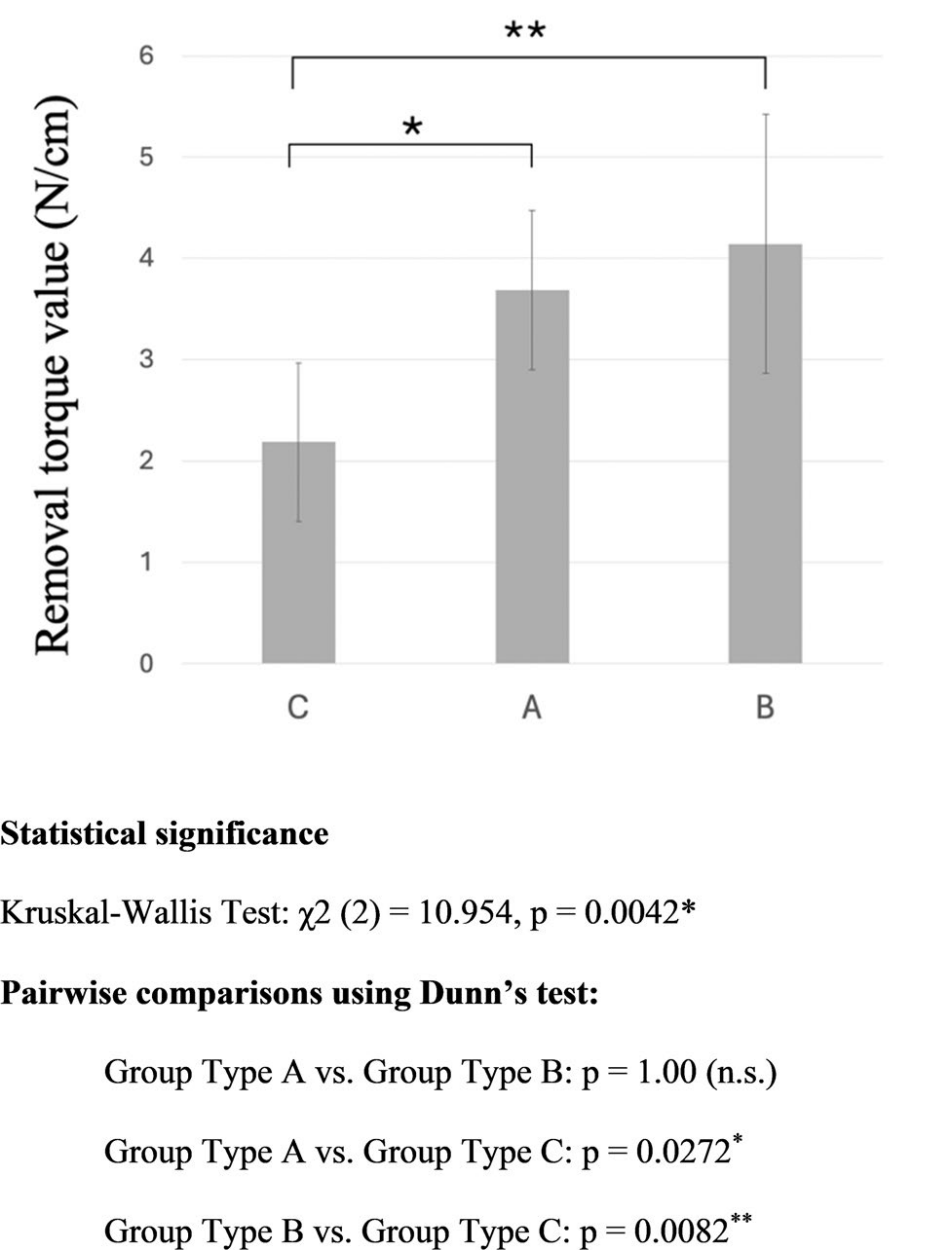
Results of the removal torque (RT) test. Results of removal torque (RT) test. A torque gauge was used to measure RT (defined as the maximum rotational load until the implant rotated horizontally). Graphs show the means and standard deviations of RT values for each group. ns: not significant

Removal torque tests revealed significant differences among the implant types. The mean removal torque values were 2.19 ± 0.78 Ncm for Type C implant group, 3.69 ± 0.78 Ncm for Type A implant group, and 4.14 ± 1.28 Ncm for Type B implant group (Fig. 4). The Type A group exhibited significantly higher removal torque values than the Type C group (*p* = 0.02), while the Type B group also had significantly higher removal torque values than the Type C group (*p* = 0.008).

### Micro-CT analysis

**Fig. 5:**
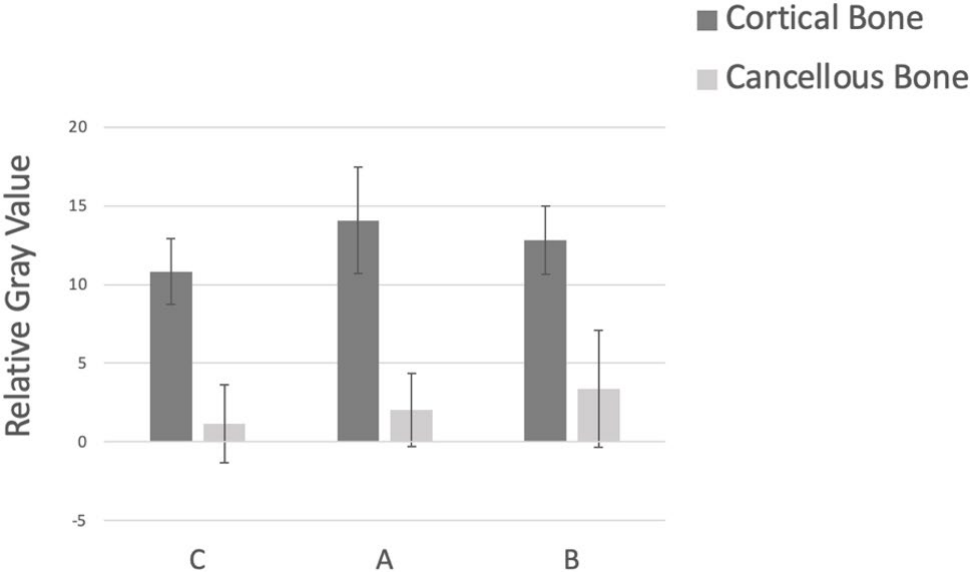
Results of micro- CT analysis. Micro-CT analysis of cortical and cancellous bone. Graphs show the means and standard deviations of the relative gray values (where water = 0 and implant = 100) for each region of interest (ROI) in each group

The average RGV of cortical bone was 14.08 ± 3.39 in the Type A group, 12.82 ± 2.16 in the Type B group, and 10.82 ± 2.09 in the Type C group. For cancellous bone, the average RGV was 2.02 ± 2.31 in the Type A group, 3.37 ± 3.72 in the Type B group, and 1.14 ± 2.47 in the Type C group. No significant differences in RGV were observed among implant types (Fig. 5).

### Histological and histomorphometric analysis

**Fig. 6:**
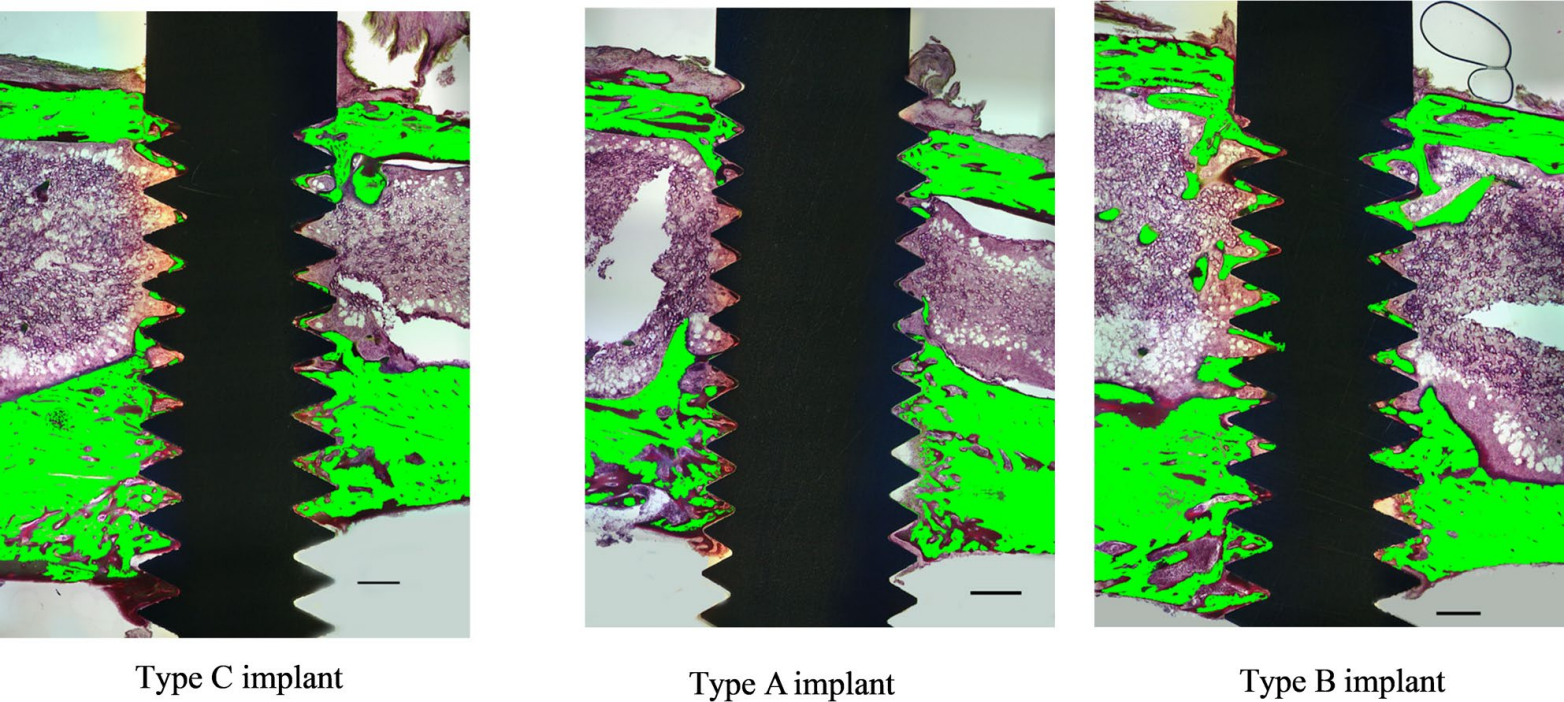
Representative histological sections for each group. Representative histological sections for each group. *Note* None of the sections intersected the fluted neck region. Scale bar: 500 μm

In the histological analysis, Fig. 6 illustrates representative sections for each group. The width of the cortical bone appeared to be similar across groups. In most cases, the osteogenic response of the peri-implant bone was more pronounced on the apical side of the implants.

**Fig. 7:**
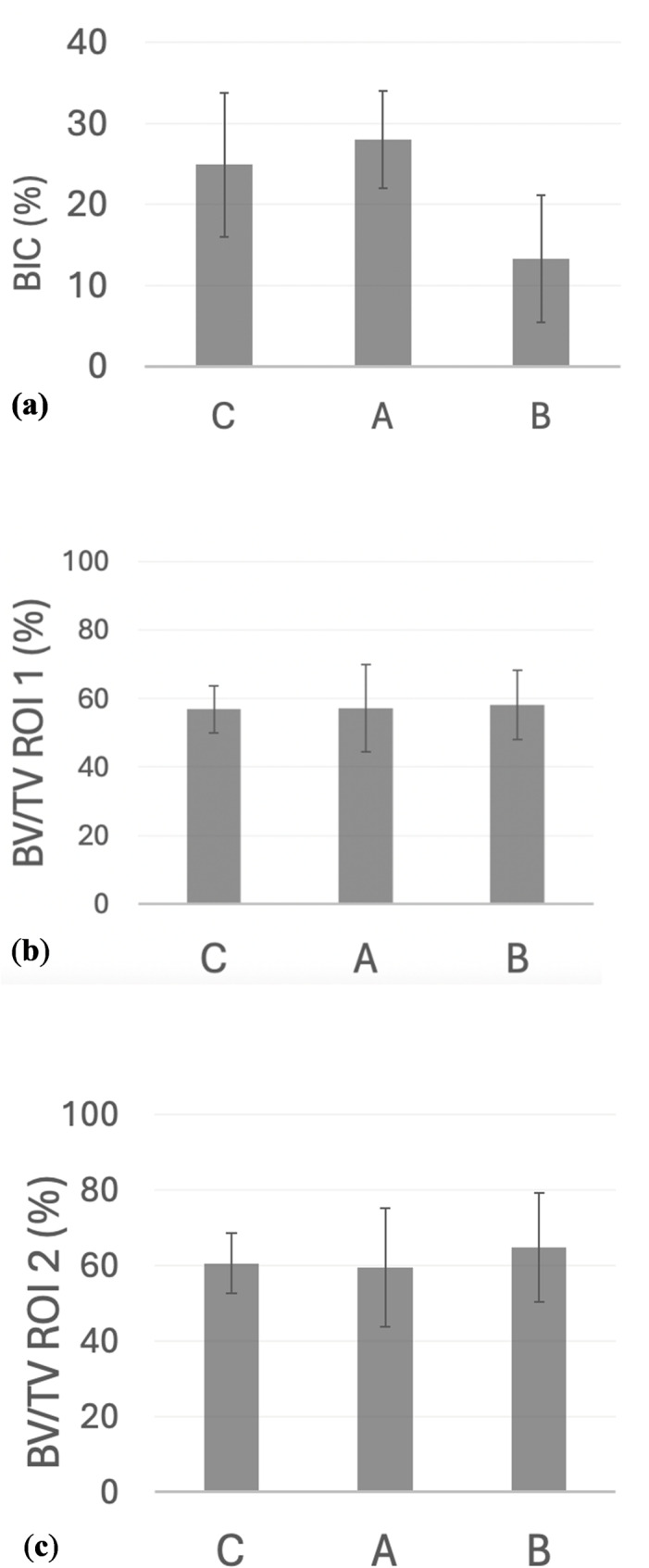
Histomorphometrical results. Histomorphometrical results for the bone-to-implant contact (BIC). **a** Means and standard deviations of the BIC for each group. Histomorphometric results of bone volume relative to tissue volume (BV/TV). Graphs show the means and standard deviations of the BV/TV at (**b**) BV/TV_ROI1 (0–100 μm from the implant surface) and **c** BV/TV_ROI2 (100–500 μm from the implant surface

In the histomorphometric analysis, the BIC tended to be higher in the Type A group compared with the Type B implant group (Fig. 7a). However, BV/TV values were not significantly different between groups (Fig. 7b, c).

## Discussion

This study evaluated the effects of coronal implant neck flutes on early peri-implant osteogenesis and stability using a rat tibial model. Implants with fluted necks (Types A and B) exhibited significantly higher removal torque values than the non-fluted control (Type C), indicating that the flute design may enhance early implant stability by influencing early peri-implant healing dynamics. Several mechanisms may underlie this improvement.

One mechanism by which the flute design may promote osteogenesis is the increased blood flow around the implant neck, which facilitates the formation of stable and wide blood clots. These clots play a crucial role in initiating bone regeneration [18, 19]. Blood components and adsorption proteins act as scaffolds linking the implant surfaces to cells, while the clot features steer the recruitment and osteogenic potential of BMSCs [16]. In particular, wider extension of the fibrin clot, which is the primary component of blood clots that adheres to the implant surface, contributes to improved healing and osseointegration [17].

Another possibility is that the flute design increases the potential contact surface for bone apposition. While these recessed areas may not directly contribute to the initial mechanical press-fit, they provide biological niches that facilitate cellular adhesion and promote mineralized tissue formation over time. In this study, Type A and Type B implants demonstrated increased calculated interface areas (1.12 mm² and 0.35 mm², respectively) compared to Type C, indicating a greater surface for bone–implant interaction.

Furthermore, the coronal flute geometry may enhance stability by creating internal spaces that act as spatial environments for bone ingrowth and mechanical interlocking [20]. These spaces are believed to contribute to the improved stability and influence the osseointegration process [21, 22]. In this study, the internal volumes of flute spaces were calculated as 0.12 mm³ for Type A and 0.57 mm³ for Type B implants. Although Type B exhibited a larger internal volume, its radiographic gray values and removal torque did not demonstrate a significant advantage over Type A, indicating that an excessive flute volume may destabilize the blood clot or interfere with effective bridging, thereby diminishing its biological and mechanical benefits.

The discrepancy observed between the removal torque and the histomorphometric results can be attributed to both methodological and biological factors. Methodologically, in this study, none of the histological sections precisely intersected the coronal flute regions due to their small size and localized positioning. All cutting planes were aligned along the long axes of the implant and tibia, which did not pass through the narrow neck-level flutes. Future studies should incorporate customized sectioning aligned with the fluted region or adopt advanced 3D imaging techniques to more accurately capture localized bone responses. Biologically, although the fluted neck provides additional surface that could support later bone apposition, such effects typically require a longer healing period for detection. At the 4-week stage, the increased removal torque in the fluted groups is therefore more likely explained by early mechanical interlocking rather than bone apposition. Further studies with extended healing times are warranted to clarify the biological contribution of the fluted design.

This study was designed as a preliminary, exploratory investigation of the effects of neck flute design on peri-implant bone healing under controlled, bacteria-free conditions. While the absence of oral microbiota in the rat tibia model prevents the evaluation of bacterial responses, this environment allows for the isolated study of the flute structure’s biological and mechanical effects without confounding infection factors. Despite this, the rat tibia remains a reliable model for implant surgery, as it has been successfully used in previous experiments [15, 23] and can be maintained without undue stress. Although the flute structure increases the BIC area and creates internal spaces that support osseointegration, it may also inadvertently create recessed pathways susceptible to bacterial colonization, thereby increasing the risk of peri-implantitis. The implant neck is a critical region that directly affects biomechanical stability and peri-implant tissue health [24, 25]. Therefore, further validation using jawbone models with natural oral flora is imperative to determine whether such design features pose microbial risks in vivo. Moreover, when applying such macrostructures to the implant neck region, it is essential to balance their biomechanical benefits with the potential biological risks.

In this study, the fluted region was intentionally positioned at or slightly above the crestal bone level to preserve a part of the structure outside the subcrestal zone to facilitate coronal bone formation. However, histological cross-sectional analysis revealed only a single instance of bone formation within a flute located above the bone crest. This finding may indicate either a limited capacity for coronal bone migration or reflect technical challenges in obtaining accurate histological sections in regions with such small, localized features.

Notably, Cohen et al. [20] introduced a similar fluted neck design; however, in their design, the flutes were positioned subcrestally, and they reported the opposite finding: flutes at the coronal region of the implant significantly impaired crestal BIC. In contrast, in the present study, we intentionally preserved a part of the flute at or slightly above the crestal level to explore whether this configuration could guide upward bone formation. Therefore, despite structural similarities, the two designs fundamentally differ in their positional rationale and underlying biological assumptions.

Future studies should aim to determine the optimal flute configuration, including size and spatial positioning, to maximize the osteogenic potential without increasing the risk of peri-implant infection. Furthermore, integrating such macro designs with antimicrobial surface modifications, improved implant–abutment interfaces, and anti-biofilm materials may enhance the long-term clinical success of these innovations.

## Conclusion

The findings of this study indicate that coronal flute designs improve implant stability and osseointegration compared with non-fluted implants. Nevertheless, further research is needed to determine the optimal flute size.

## Data Availability

The datasets generated and/or analyzed during the current study are available from the corresponding author on reasonable request.

## Abbreviations

BIC: Bone-to-implant contact
BV/TV: Bone volume relative to tissue volume
CT: Computed tomography
RGV: Relative gray value
ROI: Regions of interest
RT: Removal torque
